# Effective Concentration of Lidocaine Plus Fentanyl for Caudal Block in Patients Undergoing Transrectal Ultrasound Guided Prostate Biopsy

**DOI:** 10.1155/2016/5862931

**Published:** 2016-10-30

**Authors:** Jinguo Wang, Honglan Zhou, Wei An, Na Wang, Yang Gao

**Affiliations:** ^1^Department of Urology, The First Hospital of Jilin University, Changchun, China; ^2^Department of Anesthesiology, The First Hospital of Jilin University, No. 71 Xinmin Street, Changchun, Jilin 130021, China

## Abstract

*Objective.* This study determined the effective concentration (EC) of lidocaine plus 75 *μ*g fentanyl for caudal block in patients undergoing transrectal ultrasound (TRUS) guided prostate biopsy.* Methods.* Consecutive male patients scheduled for TRUS guided prostate biopsy were enrolled. The mixed solution for caudal block contained lidocaine and 75 *μ*g fentanyl, in total 20 mL. The concentration of lidocaine was determined using the up-and-down method, starting at 0.8% (a step size of 0.1%). A successful caudal block was defined by no pain perception during biopsy. The EC50 of lidocaine for successful caudal block was calculated and side effects were evaluated.* Results.* A total of 23 patients were recruited. The EC50 of lidocaine for successful caudal block was 0.53%.* Conclusions.* Lidocaine of 0.53% combined with 75 *μ*g fentanyl resulted in excellent caudal block in 50% of male patients undergoing transrectal ultrasound guided prostate biopsy.

## 1. Introduction

Transrectal ultrasound (TRUS) guided prostate biopsy is the standard procedure to diagnose early prostate cancer [1]. Although it is taken as a minor procedure and is always performed without anesthesia, quite a few patients still complain of pain [2, 3]. It has been reported that 19% patients would refuse to undergo a repeat biopsy without some form of anesthesia [4].

Caudal block is an established anesthesia method for urological procedures and is widely used, especially in Japan [5]. TRUS guided prostate biopsy is routinely performed in an outpatient setting. Therefore, it is important to use titrated concentrations to minimize untoward events, such as urinary retention, numbness of the lower limbs, and incapacity to ambulate. However, we have not found articles about lidocaine concentration for caudal block in patients undergoing TRUS guided prostate biopsy. Therefore, this prospective study was undertaken to investigate the effective concentration of lidocaine plus 75 *μ*g fentanyl for caudal block during TRUS guided prostate biopsy, which would be helpful as a guide for choosing an appropriate concentration.

## 2. Materials and Methods

Ethical approval of this study was provided by the institutional ethical committee. Consecutive male patients who were scheduled for TRUS guided prostate biopsy in the clinic of our department gave written informed consent participated in the study. Patients with American Society of Anesthesiologists' (ASA) physical status I or II, body mass index less than 30 kg/m^2^, normal mental status, the ability to cooperate and without previous prostate biopsies, chronic prostatitis, active urinary tract infection, inflammatory bowel disease, anorectal fissure/fistula, or allergy to local anesthetics were included.

A cleaning enema was administrated on the morning of biopsy. As antibiotic prophylaxis, intravenous 4.0 g sulbenicillin sodium or 0.2 g levofloxacin (only when the patient was allergic to sulbenicillin) was administrated one hour before the procedure. After arrival at the clinic without premedication, the patients were monitored with vital signs, and then the baselines were observed. An intravenous line was obtained in all patients and maintained with 3 mL/kg/h Ringer's lactate solution. The patients were placed in side-lying position during caudal block and biopsy. A 0.7 mm thick, 32 mm long needle was used for local anesthesia and then for caudal puncture. Lidocaine of 0.5% in a volume of 2 mL was used for subcutaneous infiltration. For caudal block, 20 mL mixed solution containing lidocaine (lidocaine, Shanghai Zhaohui Pharmaceutical Co. Ltd., Shanghai, China) of different concentrations and 75 *μ*g fentanyl (fentanyl, Yichang Humanwell Pharmaceutical Co. Ltd., Yichang, China) was administered in accordance with the up-and-down method [6, 7].

The lidocaine concentration used for each patient was determined by the outcome of the caudal block in the last patient. In the case of successful block, the concentration of lidocaine was decreased by 0.1% in the following patient. A failure caudal block was followed by a 0.1% increase in lidocaine concentration for the subsequent patient. The initial injected concentration for the first patient was 0.8% based on our clinical experience with the method. A negative aspiration test along with 5 mL test bolus of the caudal solution was performed before injection of the residual volume in order to avoid intravascular or subarachnoid injection. The injection was along with repeated intermittent aspirations. If three attempts of caudal puncture failed, caudal block was given up and the patient was excluded from the study. Ten minutes later, preliminary digital rectal examination was done before insertion of the transrectal probe (PVT-781-BT, Toshiba, Nasu, Japan) with lubrication of ultrasound gel (Bailesi, Tianjin Xinyan Medical Equipment Co. Ltd., Tianjin, China), and then prostate biopsy was performed using a BARD Magnum biopsy gun (MG1522, Bard Company, Covington, US).

A successful caudal block was defined as no pain perception during prostate biopsy. The caudal block was considered a failure if the patient complained of pain during prostate biopsy. The biopsy and caudal block were performed by the same urologist and anesthesiologist, respectively. The urologist and patients were kept blinded to the lidocaine concentration. Hypotension was defined as a 20% decrease of systolic pressure from the baseline (the mean of two values measured noninvasively in the supine position on arrival at the clinic). Bradycardia was defined as a heart rate <50 beat/min. After biopsy, motor block was assessed. All patients were observed for 1 hour after cystoscopy before they went home.

The primary outcome was the EC50 of lidocaine. The up-and-down method needed at least six success-failure pairs for statistical analysis. The number of patients had to be increased if fewer than six success-failure pairs appeared [6, 7].

For statistical analyses, SPSS 21.0 (SPSS Inc., Chicago, IL, USA) was used. The demographic data, the procedural data, and hemodynamic values were represented as number of patients or mean ± standard deviation. The EC50 of lidocaine for successful caudal block was calculated using Dixon's up-and-down method [6–8]. Hemodynamic changes were analyzed using repeated measures ANOVA within each group and unpaired *t*-test between the two groups. A *P* value of less than 0.05 was considered to be statistically significant.

## 3. Results

Twenty-five patients were recruited and 23 completed the study. One patient was excluded from the study because of a failure of caudal puncture; the other was excluded because of refusal to cooperate. Twenty-three patients were analyzed to obtain six pairs of success-failure combinations. The demographic and the procedural data were listed in Table 1.

**Figure 1:**
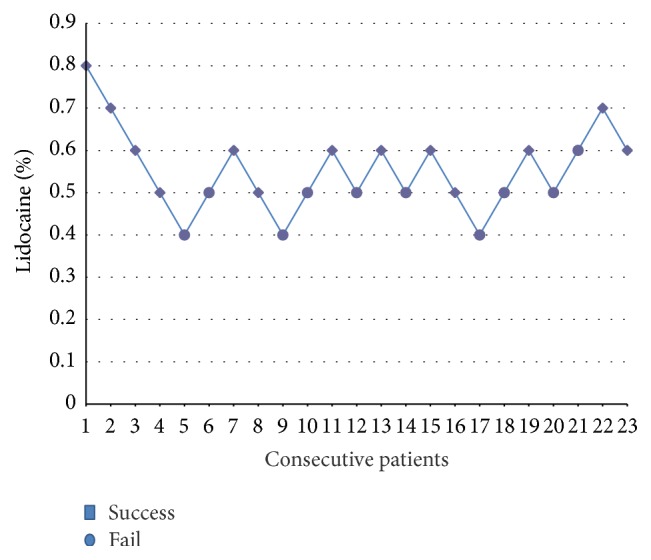
The anesthetic effect of different lidocaine concentrations for caudal block in 23 consecutive patients undergoing TRUS guided prostate biopsy.

Figure 1 shows the six up-and-down pairs of concentrations of lidocaine. As a result, the patients received 0.8 (*n* = 1), 0.7 (*n* = 2), 0.6 (*n* = 8), 0.5 (*n* = 9), or 0.4 (*n* = 3)% of lidocaine. Using Dixon's up-and-down method, EC50 of lidocaine was 0.53%.

Mean blood pressure (MBP) and hear rate (HR) were significantly increased at 2 minutes after the first shot of biopsy compared with the baseline within the fail group. No significant difference was found in MBP and HR between the success group and the fail group at 1 and 2 minutes after the first shot of biopsy (Table 2). Hypotension or bradycardia was not observed in any patient.

The lidocaine concentrations used in these patients ranged from 0.4% to 0.8%. No side effects associated with caudal block were recorded in these patients. No postoperative ambulation with support was observed.

## 4. Discussion

Outpatients are considered to be more vulnerable to the adverse effects, especially motor blockade and urinary retention, so we estimated the effective dose for adequate anesthesia with low possibility of adverse effects in this group of patients. Accordingly, the estimated effective concentration of lidocaine would be meaningful for clinical practice. In the current study, the minimal concentration of lidocaine plus 75 *μ*g fentanyl for successful caudal block during TRUS guided prostate biopsy is determined. The EC50 values of lidocaine for caudal block are 0.53%.

The safety and efficacy of caudal block for perineal surgeries have been reported in adults [9–14]. In terms of clinical aspects, we consider that it is desirable to obtain adequate anesthetic conditions within short time interval without influence on ambulation. Lidocaine is cheap, safe, and easily available and has a fast onset and short duration of action, so it is suitable for outpatient anesthesia. Fentanyl as an adjuvant to local anesthetics can decrease the dose of local anesthetics and then the level of motor block, improve the quality of anesthesia, and extend the duration of analgesia [12–14]. In this study, all patients can walk without assistance after the procedure which is beneficial for the outpatient procedures.

The result of the present study is consistent with the studies conducted by Cesur et al. and Ikuerowo et al. [13, 14]. The authors reported significantly decreased pain in the caudal group compared with the gel group. As an additional advantage, caudal block provides sphincter relaxation, making it much easier to manoeuvre the TRUS probe.

Horinaga et al. reported that caudal block was less effective than periprostatic nerve block [15]. In their study, the local anesthetic solution for caudal injection is only 10 mL, which is a very low volume for caudal anesthesia. A successful caudal anesthesia need two factors: enough volume and adequate concentration. For sacral anesthesia, at least 15 mL of local analgesics is needed [16, 17]. We chose a dose of 20 mL for caudal injection, according to the study of Li et al. and Cesur et al. [12, 13].

According to the up-and-down method, EC50 can be obtained with as few as one-fifth of the number of patients as the traditional design with a preset number of patients at each level, which means that we can minimize the number of patients given insufficient treatment [6]. The result of this study indicates that MBP and HR are significantly increased at the time point of 2 minutes after the first shot of biopsy compared with the baseline within the fail group. It suggests that it is very necessary to decrease the number of patients given insufficient anesthesia.

There are several limitations of the present study. The EC50 value provided in this study is applicable only when 75 *μ*g fentanyl is combined. With the difference in the combined drug or dose, the EC50 value should be changed. In addition, the findings of our study are applicable to male patients only. Further studies will focus on sex differences in effective concentrations of lidocaine for caudal block.

In conclusion, we suggest that the EC50 value of lidocaine for caudal block in patients undergoing TRUS guided prostate biopsy is 0.53%.

## Figures and Tables

**Table 1:** The demographic and the procedural data.

Age (yr)	65.3 ± 9.3
Weight (kg)	60.3 ± 8.5
ASA I/II (*n*)	15/8
The procedure duration (min)	6.8 ± 2.4
Body mass index (kg/m^2^)	24.3 ± 2.4

Data are presented as mean ± standard deviation or number of patients. ASA: American Society of Anesthesiologists.

**Table 2:** MBP and HR at various time points.

		*T*0	*T*1	*T*2	*P* value
MBP	The success group (*n* = 13)	95.2 ± 9.1	97.5 ± 10.2	98.9 ± 8.3	0.591
	The fail group (*n* = 10)	94.6 ± 8.7	99.3 ± 9.7	105.3 ± 8.9	0.045
	*P* value	0.874	0.672	0.09	
HR	The success group (*n* = 13)	69.2 ± 7.8	71.2 ± 8.8	73.1 ± 8.6	0.503
	The fail group (*n* = 10)	68.7 ± 8.1	73.5 ± 7.7	79.1 ± 9.2	0.033
	*P* value	0.882	0.519	0.122	

Data are presented as mean ± standard deviation or number of patients. *T*0: the average of two consecutive invasive blood pressures in the supine position on admission to the clinic room; *T*1: 1 minute after the first shot of biopsy; *T*2: 2 minutes after the first shot of biopsy; MBP: mean blood pressure; HR: heart rate.
